# ERP markers of target selection discriminate children with high vs. low working memory capacity

**DOI:** 10.3389/fnsys.2015.00153

**Published:** 2015-11-05

**Authors:** Andria Shimi, Anna Christina Nobre, Gaia Scerif

**Affiliations:** ^1^Attention, Brain, and Cognitive Development Lab, Department of Experimental Psychology, University of OxfordOxford, UK; ^2^Brain and Cognition Lab, Oxford Centre for Human Brain Activity, Department of Psychiatry, University of OxfordOxford, UK

**Keywords:** selective attention, encoding, visual working memory, development, ERPs, contralateral posterior negativity, N2pc

## Abstract

Selective attention enables enhancing a subset out of multiple competing items to maximize the capacity of our limited visual working memory (VWM) system. Multiple behavioral and electrophysiological studies have revealed the cognitive and neural mechanisms supporting adults’ selective attention of visual percepts for encoding in VWM. However, research on children is more limited. What are the neural mechanisms involved in children’s selection of incoming percepts in service of VWM? Do these differ from the ones subserving adults’ selection? Ten-year-olds and adults used a spatial arrow cue to select a colored item for later recognition from an array of four colored items. The temporal dynamics of selection were investigated through EEG signals locked to the onset of the memory array. Both children and adults elicited significantly more negative activity over posterior scalp locations contralateral to the item to-be-selected for encoding (N2pc). However, this activity was elicited later and for longer in children compared to adults. Furthermore, although children as a group did not elicit a significant N2pc during the time-window in which N2pc was elicited in adults, the magnitude of N2pc during the “adult time-window” related to their behavioral performance during the later recognition phase of the task. This in turn highlights how children’s neural activity subserving attention during encoding relates to better subsequent VWM performance. Significant differences were observed when children were divided into groups of high vs. low VWM capacity as a function of cueing benefit. Children with large cue benefits in VWM capacity elicited an adult-like contralateral negativity following attentional selection of the to-be-encoded item, whereas children with low VWM capacity did not. These results corroborate the close coupling between selective attention and VWM from childhood and elucidate further the attentional mechanisms constraining VWM performance in children.

## Introduction

Temporary storage of information is essential in order to act on our ever-changing visual world. However, our visual working memory (VWM), the system responsible for keeping information in an “on-line” state, is highly limited to about four items (Cowan, 2001; Todd and Marois, 2004). Yet, at any given moment we are faced with multiple items competing for representation. To maintain an adaptive behavior, we need to represent only the most relevant information at any time. Visual selective attention allows us to select and process the items that are most relevant to current goals by shifting our focus to locations or objects. Influential theories of attention have postulated that a key basic mechanism to resolving the competition among competing items is *selectivity*, the ability to attend to the most relevant information and ignore the irrelevant (e.g., Desimone and Duncan, 1995; Desimone, 1998; Corbetta et al., 2000; Kastner and Ungerleider, 2001).

Indeed, over the last decade, research findings have not only highlighted the dynamic interplay between selective attention and VWM (Corbetta et al., 2002; Mayer et al., 2007; Chun and Johnson, 2011; Ikkai and Curtis, 2011; Fusser et al., 2012; Gazzaley and Nobre, 2012; Cohen et al., 2014) but critically, have demonstrated that individual differences in the efficiency of selective attention underpin differences between individuals with high vs. low VWM capacity, both in young and late adulthood (Vogel and Machizawa, 2004; Gazzaley et al., 2005; Vogel et al., 2005; McNab and Klingberg, 2008; Jost et al., 2011; Linke et al., 2011). Taken together, these findings have shown that the mechanisms responsible for the selection and encoding of representations into VWM underpin efficient storage and higher VWM capacity.

VWM increases dramatically with age (Gathercole, 1999; Cowan et al., 2005) with accompanied maturational changes in the brain (Kwon et al., 2002; Klingberg et al., 2002; Luna et al., 2004; Crone and Ridderinkhof, 2011; Jolles et al., 2011; Barriga-Paulino et al., 2014). Driven by the advances in the adult cognitive neuroscience literature and given that selective attention also undergoes dramatic improvement during childhood (Plude et al., 1994; Scerif, 2010; Johnson, 2011; Stevens and Bavelier, 2012), recent developmental research has also started examining the influence of visual attention mechanisms on the developing VWM system (Olesen et al., 2007; Cowan et al., 2010; Ross-Sheehy et al., 2011; Sander et al., 2011; Wendelken et al., 2011; Astle et al., 2012, 2014; Markant and Amso, 2013; Shimi et al., 2014a,b; Shimi and Scerif, 2015), rather than focusing solely on increases in VWM storage. Extending the adult findings to the developmental domain, in a recent study, Shimi et al. (2014a) demonstrated that age-related differences in the temporal dynamics of attentional orienting mechanisms before or after encoding items in VWM contributed to differences in VWM performance between children and adults. Importantly, individual differences in the temporal dynamics of the preparatory attentional orienting mechanisms that *bias the encoding* of relevant items into VWM discriminated children with high vs. low VWM capacity.

Despite this growing body within developmental science, our knowledge about the interactions between selective attention and VWM in children remains primarily focused on behavioral performance, rather than on the underlying neural circuits. Multiple electrophysiological studies have investigated the neural mechanisms supporting adults’ selective attention of incoming percepts in function of encoding in VWM. However, an understanding of analogous processes in children is significantly more limited. Similarly, knowledge about children’s selective attention in service of VWM is disproportionally limited compared with knowledge about selective attention for sensory processing. In the sensory, rather than the memory domain, research has shown that the speed and the efficiency of the ability to select the relevant stimulus among competing items improves with age, possibly reflecting the protracted development of neural networks controlling selective attention (for reviews, see Ridderinkhof and van der Stelt, 2000; Stevens and Bavelier, 2012). Thus, here, we examined whether the neural correlates of attentional selection during encoding into VWM operate differently in childhood compared to adulthood. In addition, we examined whether individual differences in children’s *efficiency of attentional selection* of the relevant item during encoding relates to individual differences in VWM capacity. This is typically assessed in more traditional behavioral terms, through explicit recognition memory at the end of the trial sequence. However, the event-related brain potentials (ERP) method can track electrical brain responses on a millisecond-by-millisecond resolution, and it is therefore ideally suited for investigating attentional mechanisms leading to later accurate memory at different processing stages (Hillyard and Anllo-Vento, 1998; Luck et al., 2000), in both children and adults. This high temporal resolution has important implications for increasing an understanding of the neural mechanisms supporting VWM across development. For example, we have previously seen that neural activity elicited when guiding attention to a location of an upcoming target via an attentional cue, i.e., an initial stage within the information processing stream [as reflected in ERP components such as the Early Directing Attention Negativity (EDAN), the Anterior Directing Attention Negativity (ADAN), and the Late Directing Attention Negativity (LDAP)], differed not only between children and adults, but also between children of high vs. low VWM capacity (Shimi et al., 2014a). Here, we asked the following complementary question: Do age group and individual differences in neural activity hold for a subsequent stage within the information processing stream, i.e., selecting efficiently the relevant to-be-encoded item in VWM?

We examined this question by focusing on a well-known lateralized electrophysiological marker of attentional selection of a target item among multiple competing items, the N2pc (Luck and Hillyard, 1994a,b; Eimer, 1996; Hickey et al., 2009). N2pc is an enhanced negativity elicited over posterior scalp sites contralateral to the side of the attended item, typically within the latency range of ~200–350 ms post-stimulus. N2pc has been heavily studied within the adult population in the sensory domain (Woodman and Luck, 1999; Hopf et al., 2004; Kiss et al., 2008; Mazza et al., 2009; Woodman et al., 2009) and more recently in the VWM domain (Nobre et al., 2008; Astle et al., 2009; Kuo et al., 2009; Shimi et al., 2014a). In contrast, in children, studies of N2pc are exceptionally scarce: to our knowledge, only two studies have examined N2pc in typically developing children to date. One of these studies investigated the selection of sensory targets among distractors using a visual search paradigm (Couperus and Quirk, 2015), and the other study found the N2pc to be elicited by children when they retrospectively searched their VWM (Shimi et al., 2014a); in both cases, similarities and differences emerged between children and adults in the topography and latency of the N2pc respectively. Thus far, no published study has examined whether N2pc is involved in attentional selection during the encoding of information in VWM in childhood; and if so, whether it resembles the spatiotemporal characteristics of the N2pc involved in attentional selection during VMW encoding in adulthood. Here, by measuring N2pc, we examined: (1) whether children and adults elicit similar neural activity when selecting a target item among competing items, for encoding in VWM and (2) whether this neural activity relates to individual differences in VWM capacity in children.

## Materials and Methods

### Participants

Seventeen typically developing children (5 males and 12 females), aged 10–11 years old (*M* = 10.2 years old, *SD* = 0.39), and 15 healthy adults (8 males and 7 females), 21–34 years old (*M* = 26.4 years old, *SD* = 3.76), participated in the study. Children were recruited from local primary schools via an opt-in procedure and adults were recruited among University postgraduate students. All participants were right-handed and had normal or corrected-to-normal vision. The study had ethical approval from the Central University Research Ethics Committee of the University of Oxford. Prior to testing, adult participants and parents of child participants signed a consent form whereas children assented to participate in the study verbally. For their participation, adults received monetary compensation and children received an appreciation certificate. One adult participant was excluded from the analyses due to significantly below-chance behavioral performance. The same sample of participants was included in complementary analyses to those reported here, that focused on activity associated with attentional cues, rather than VWM arrays (Shimi et al., 2014a). We chose 10–11 year-olds as our age-comparison group to the adult group because a few developmental studies have shown that some cognitive control abilities reach the adult mature state around the age of 10–11 years of age whereas other cognitive control abilities continue to develop until later in adolescence (e.g., Huizinga et al., 2006). Based on this, 10–11 year-olds could either be similar to adults or still developing, making them thus an interesting target age group to study the developmental state of selective attention and WM processes. Also, taking into account the large variability that may exist in children’s data, we opted for a narrow age group that would provide more statistical power and maximize the likelihood of separating age-related and individual differences.

### Task and Stimuli

**Figure 1:**
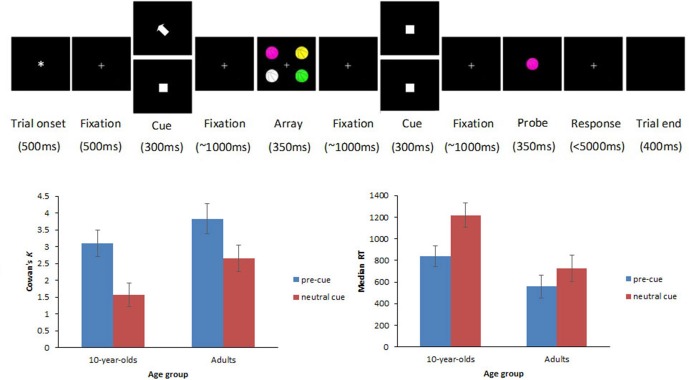
**Top row illustrates an example trial sequence.** Each trial began with an asterisk (500 ms), followed by a fixation point which remained visible throughout the trial. Five hundred millisecond later, a cue appeared for 300 ms. In cued trials, an arrow pointed to the item that participants should encode in visual working memory (VWM; top display at the cue position), whereas in neutral trials, the cue was replaced by a spatially uninformative white square (bottom display at the cue position). After a randomly varied fixation interval (800–1200 ms), the memory array with the four colored items appeared for 350 ms, followed by a randomly varied fixation interval (800–1200 ms). Depending on the trial type, participants had to encode in VWM either one item out of the four (cued trials) or all four items (neutral trials). Subsequently, another spatially uninformative white square stimulus appeared for 300 ms. After a randomly varied fixation interval (800–1200 ms), the probe appeared for 350 ms followed by a fixation point that remained on the screen until a response was made or until a maximum of 5000 ms elapsed (leading to minimal trial attrition across age-groups). Participants had to respond whether the probe was present in the array or not by pressing mouse buttons. Bottom row shows Cowan’s *K* (left panel) and median RT (right panel) scores on cued and neutral trials for 10-year-olds and adults. Error bars represent ±95% confidence intervals.

The full study design was described in detail elsewhere (Shimi et al., 2014a). Here, we describe only the trial types related to the focus of the current paper. These are illustrated in Figure 1. Participants viewed arrays of four colored items, followed by a single colored probe item after a variable delay. They were instructed to indicate whether the probe was present among the initial four items by pressing a mouse button (left for present and right for absent). Items comprised identical line drawings of familiar objects and cartoons (e.g., basketballs, each subtending 1.64° × 2.05° of visual angle from a distance of 100 cm and centered at 2.87° lateral and 2.87° azimuthal eccentricity from a central fixation point). The items were presented in different colors (drawn from a set of seven colors: white, red, magenta, orange, yellow, green, and blue) on a black background. On half trials, the memory array was preceded by a spatial cue (white arrow; 0.82° × 0.82°) that guided the participants’ attention to one of the upcoming items of the array and was fully informative (100%) of the location of a target probe, should this appear in the memory array (cued trials henceforth). The cue was equally likely to point to one of the four possible locations. On the other half trials, a spatially uninformative white square (0.82° × 0.82°) was presented before the array (neutral trials henceforth), and served the purpose of controlling for the non-spatial alerting effects that the spatial cue may engender.

Participants completed 12 practice trials to familiarize themselves with the task, followed by 192 test trials divided into blocks of 48 trials in each, with 67% of trials containing the probe in the memory array (“probe present”) and 33% of trials not containing it (“probe absent”). Cued and neutral trials were intermixed randomly within each block.

### Procedure

Participants were comfortably seated in a dimly illuminated, electrically shielded room, and were given written and verbal instructions along with examples on cards. On practice trials, participants received verbal feedback from the experimenter and visual feedback (correct, incorrect, no response) on the screen after each trial, whereas on test trials, participants received feedback about the number of correct responses every 16 trials and at the end of each block. Participants were recommended and reminded prior to the beginning of each block to pay attention to the cue as it would help them decide whether the probe item reappeared. They held the mouse with their right hand and were advised to respond as quickly and accurately as possible while maintaining their gaze on the fixation point throughout the trial. They were also asked to blink as little as possible, preferably after they responded, and to try to remain still during task performance. Participants were monitored throughout the task via a camera to ensure that they were engaged in the task and that they were not moving or blinking excessively during the task. All participants completed all test blocks except one child that completed one block less due to fatigue and loss of interest to the task. Self-paced breaks were inserted between blocks.

### EEG Recording and Processing

EEG was recorded continuously using a NuAmp amplifier (Neuroscan, Inc.) from 19 silver/silver chloride electrodes mounted on an elastic cap and positioned according to the International 10–20 system (AEEGS, 1991). The montage included four midline scalp sites (Fz, FCz, Cz, Pz) and five scalp sites over each hemisphere (F3/F4, C3/C4, P3/P4, PO7/PO8, O1/O2). Additional electrodes were used as ground and reference sites. The electrode placed at AFz on the midline served as the ground. The EEG was referenced on-line to the FCz electrode and then re-referenced off-line to the algebraic average of the left and the right mastoids. Blinks and eye movements were monitored by deriving bipolar recordings from electrodes placed on the outer canthi of both eyes (HEOG) and from one electrode placed below the right eye and F4 (VEOG). Electrode impedances were kept below 5 kΩ. The ongoing brain activity at all scalp sites was sampled every 1 ms (1000 Hz analog-to-digital sampling rate) and filtered with a band-pass of 0.50–70 Hz.

The EEG data were then filtered off-line with a low-pass filter of 40 Hz and the continuous EEG was segmented into epochs, time-locked to the onset of the memory array in cued trials. Given that we were interested in neural activity that was lateralized with respect to the side of the to-be-encoded item, epochs from leftward and rightward trials were combined with an averaging procedure that preserved the spatial location of the electrodes relative to the position of the to-be-encoded item (i.e., contralateral or ipsilateral). Epochs started 100 ms prior to- and ended 600 ms after stimulus onset. ERP amplitude values were baseline corrected relative to a −100–50 ms stimulus interval. Epochs containing excessive noise or drift (±100 μV for adults and ±150 μV for children) at any electrode were excluded from subsequent analyses. Furthermore, epochs containing blinks or eye movements (±50 μV for adults and ±100 μV for children) were rejected. The thresholds for each age-group were chosen based on previous ERP parameters used with adults (e.g., Murray et al., 2011) and with children (e.g., Melinder et al., 2010) and to be in line with the previous ERP parameters used with the same sample of participants (Shimi et al., 2014a). Due to skull differences (Scerif et al., 2006) as well as other physiological differences between children and adults (e.g., brain tissue) and given that children’s spectral power is higher than adults’ (Barriga-Paulino et al., 2011), different artifact rejection thresholds are required in order to refrain from excluding clean EEG trials from the children’s data. In addition, all epochs were visually inspected for any residual artifacts, which were all manually eliminated, an additional check that was especially important for lateralized eye-movements, as these may capture overt rather than covert attention. This artifact rejection procedure resulted in retaining approximately 82% of overall trials for adults and 85% of overall trials for children. Finally, trials with incorrect behavioral responses were discarded. In order to maintain an acceptable signal-to-noise ratio, the accepted lower number of trials per participant was set to 20 trials, and on average retained 70 trials for adults and 74 trials for children.

### ERP Analyses

The aim of this experiment was to examine children’s neural correlate of selecting one out of multiple items for encoding into VWM, its relation with behavior, and whether it resembles the neural correlate observed in adults. Hence, the ERP analyses focused on epochs locked to the memory arrays presented after an attentional cue guided participants’ attention to the item that they should encode in VWM. We targeted a well-known lateralized ERP marker of attentional selection, namely N2pc, and we quantified it as the mean voltage difference between contralateral and ipsilateral sites relative to the side of the to-be-selected item (target henceforth). Based on the previous findings, N2pc was expected to occur, and therefore measured, at posterior electrodes, PO7/8 and O1/2. We examined the presence of N2pc only for memory arrays in cued trials as there was not one specific lateralized item to be encoded in arrays of neutral trials, rather participants had to encode all four items. The time windows for analyzing the N2pc for each age group were selected on the basis of the following latency analysis: lateralized voltage differences were tested in successive time-bins in steps of 40 ms intervals between 260 and 400 ms following visual inspection of the two group average waveforms. Effects were considered significant if a *p* < 0.05 criterion was exceeded for 40 ms and persisted over at least two successive time bins in a given region. This exploratory analysis for each age group guided the selection of the time window with which to test for the presence of an N2pc effect. A two-way repeated measures ANOVA was then conducted on the mean amplitude of the neural activity in the longer time window merging the time-bins in which the effects were found significant and sustained, testing the effects of electrodes (PO7/8 and O1/2) and visual hemifield (contralateral and ipsilateral to the target).

### Behavioral Analyses

Separate mixed-design ANOVAs were performed on d-prime, *K*, and median RT scores with trial type (cued, neutral) as the within-subject variable and the age group (10-year-olds, adults) as the between-subject variable. D-prime and Cowan’s *K* measures converged so for brevity here we report statistics only for *K*. Cowan’s *K* is a memory capacity measure that reflects the number of stored items in memory (Pashler, 1988; Cowan, 2001) and here was calculated using the formula: *K* = S (set size of the initial array) × (hit rate − false alarm rate). Hit rate was defined as the conditional probability that the participants responded probe present when the probe was indeed present and false alarm rate was defined as the conditional probability that the participants responded probe present when in fact the probe was absent. Extreme scores (e.g., perfect hit rate) were adjusted using the formula 1−(1/2N) as recommended by Macmillan and Creelman (2005) where N = the number of total trials in a condition. RTs were computed for probe-present trials and for correct responses only because incorrect responses and absent trials maybe influenced by multiple non attentional processes (as discussed in Griffin and Nobre, 2003). In addition, we explored functional links between behavioral performance and neural activity in children via split-half paired-sample *t*-tests on high- and low- memory capacity groups (as a function of cueing benefit in *K*) separately.

## Results

### Behavioral Results

There were significant main effects of age group, *F*_(1,29)_ = 14.65, *p* = 0.001, with overall higher *K* scores in adults (*M* = 3.24) compared with children (*M* = 2.33), and trial type, *F*_(1,29)_ = 96.41, *p* < 0.001, with significantly higher *K* scores in cued (*M* = 3.46) than in neutral trials (*M* = 2.11). The interaction of age group × trial type did not reach significance, *F*_(1,29)_ = 1.82, *p* = 0.19, suggesting that benefits from cues in accuracy did not differ significantly between children and adults.

The analysis on median RTs to probes accurately reported as present in the memory array showed significant main effects of age group, *F*_(1,29)_ = 35.27, *p* < 0.001, and trial type *F*_(1,29)_ = 51.72, *p* < 0.001, as well as a significant interaction of age group × trial type, *F*_(1,29)_ = 7.47, *p* = 0.011. Analyses of simple main effects for the age-group × trial type interaction revealed that the interaction was driven by a smaller RT benefit drawn from cues by adults (*M* = 170) than children (*M* = 378, *p* = 0.008). A subsequent difference-scores analysis was carried out to interpret the interaction independently of baseline differences on neutral trials, and taking overall slowing in RT into account by treating RT differences as proportions of neutral RTs [(neutral-cued)/neutral]. The effect on scaled RTs did not remain significant (*p* = 0.25), thus suggesting that the larger RT benefits in children depended on overall slowing in baseline responses by the children. Figure 1 shows behavioral results.

### ERP Results

#### Adults

**Figure 2:**
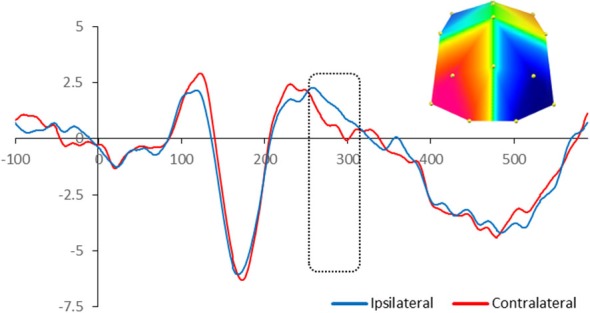
**Grand-averaged waveforms for N2pc elicited by the memory array in cued trials in adults.** Red lines indicate neural activity contralateral to the side of the to-be-encoded item and blue lines indicate neural activity ipsilateral to the side of the to-be-encoded item. Positive voltage is plotted upwards. The dotted box highlights the time-window during which the mean voltage difference of the N2pc was found significant. The topographic map next to the ERP waveform panel shows the lateralized difference in voltage between contralateral and ipsilateral sites during the time window in which the N2pc component was found significant. The voltage distributions are shown from posterior perspective. Blue indicates negative voltage and red indicates positive voltage.

For adults, there was significant enhanced negativity contralateral to the position of the target in the memory array between 260 and 320 ms at PO7/8 and O1/2 sites, *F*_(1,13)_ = 6.03, *p* = 0.029, reflecting the N2pc. Figure 2 illustrates the neural activity elicited during attentional selection of the target item for encoding into VWM for adults.

#### Children

**Figure 3:**
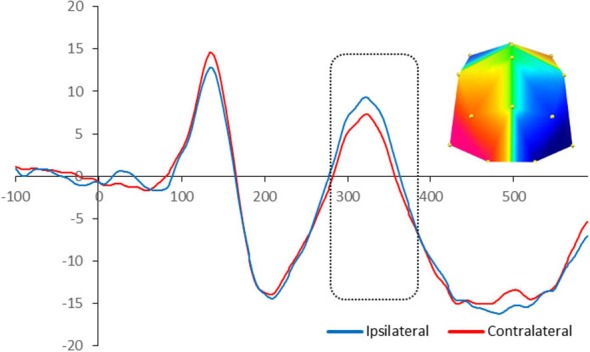
**Grand-averaged waveforms for N2pc elicited by the memory array in cued trials in 10-year-olds.** Red lines indicate neural activity contralateral to the side of the to-be-encoded item and blue lines indicate neural activity ipsilateral to the side of the to-be-encoded item. Positive voltage is plotted upwards. The dotted box highlights the time-window during which the mean voltage difference of the N2pc was found significant. The topographic map next to the ERP waveform panel shows the lateralized difference in voltage between contralateral and ipsilateral sites during the time window in which the N2pc component was found significant. The voltage distributions are shown from posterior perspective. Blue indicates negative voltage and red indicates positive voltage.

The statistical analysis on the children’s ERP amplitude showed similarities and differences compared to adults in terms of topography of the effects and their timing respectively. There was significant enhanced negativity contralateral to the position of the target in the memory array between 280 and 380 ms at PO7/8 and O1/2 sites, *F*_(1,16)_ = 4.74, *p* = 0.045, signifying N2pc. Figure 3 illustrates the neural activity elicited during attentional selection of the target item for encoding into VWM for children.

### Electrophysiological Predictors of VWM Capacity in Children

Subsequently, we examined whether children’s ability to deploy attentional selection in function of encoding into VWM related to their VWM capacity. We chose to examine this in the time-window that the N2pc was elicited in adults (i.e., 260–320 ms), in order to investigate whether children that demonstrate a magnitude of “adult-like” neural activity during attentional selection at encoding, will show a greater cueing benefit in VWM capacity. Previous results have shown that the large variability in children’s VWM capacity is explained by some children demonstrating an “adult-like” neural profile in their efficiency of preparatory attention whereas other don’t (Shimi et al., 2014a). By examining a similar question here, results can demonstrate functional links between the efficiency of attentional selection at encoding and later VWM performance in childhood, a question that has not been investigated before.

**Figure 4:**
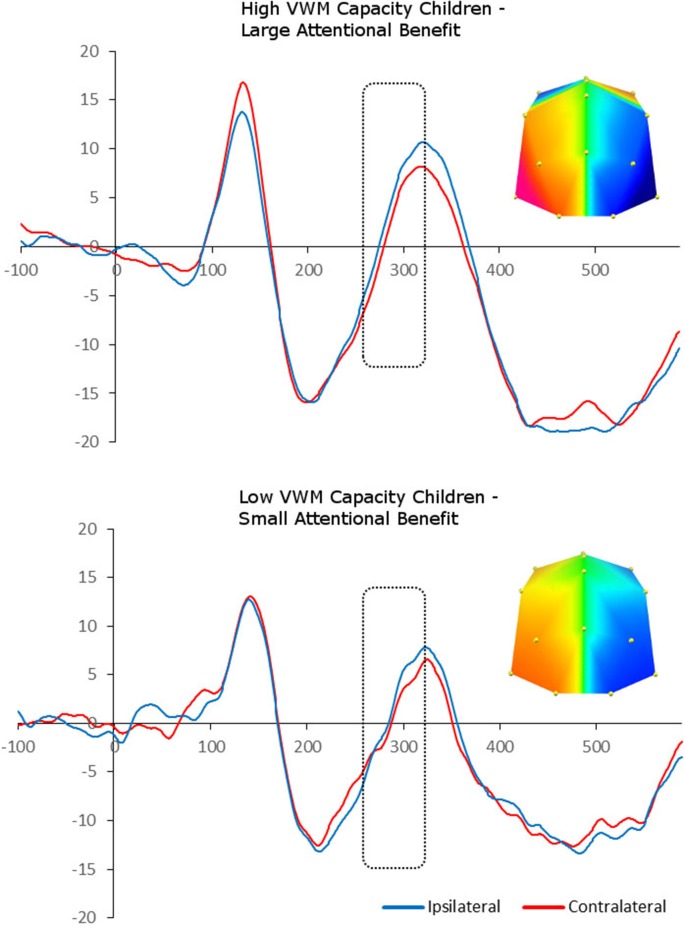
**Grand-averaged waveforms for N2pc elicited by the memory array in cued trials and divided between high- and low-memory capacity children (on the basis of *K* benefit).** Red lines indicate neural activity contralateral to the side of the to-be-encoded item and blue lines indicate neural activity ipsilateral to the side of the to-be-encoded item. Positive voltage is plotted upwards. The topographic maps next to the ERP waveform panels show the lateralized difference in voltage between contralateral and ipsilateral sites during the “adult time window” in which the N2pc component was found significant (260–320ms). The voltage distributions are shown from posterior perspective.

We carried out median-split analyses, by dividing children into high- and low-capacity groups (on the basis of *K* benefit). This allowed us to carry out paired-sample *t*-tests between contralateral and ipsilateral ERP amplitudes, and therefore explore the presence of “adult-like” N2pc in each capacity group separately. Splitting the children into those who showed a large vs. small cue benefit following spatial cues in terms of *K* revealed a significant enhanced negativity contralateral to the position of the target in the memory array between 260 and 320 ms at PO7/8 and O1/2 sites, *F*_(1,8)_ = 5.77, *p* = 0.04, i.e., N2pc, for the large cue benefit group. In contrast, there was no statistically significant N2pc in the small cue benefit group, *F*_(1,7)_ = 0.27, *p* = 0.62 (Figure 4).

## Discussion

The aims of this study were to identify the ERP correlates of children’s attentional selection of a target item, among multiple competing items, during encoding in VWM, and to test whether these resemble the neural correlates involved in adults’ selective encoding in VWM. Results showed that both children and adults elicited a significant negativity contralateral to the item to be encoded in VWM, i.e., both age groups elicited an N2pc. The observed ERP component had a similar topographical distribution between the two age groups but differed in latency. Importantly, individual differences in the extent to which the N2pc at encoding was “adult-like” related to variation in VWM performance at the end of the trial in children.

Despite overall better VWM performance and higher VWM capacity for adults compared to children, all participants benefitted from cues before encoding. This suggests that when the memory array appeared, both children and adults largely selected the item to be probed for encoding in VWM. This behavioral finding was corroborated by the neural activity participants elicited following memory array onset: all participants elicited greater negativity at posterior scalp sites (O1/2 and PO7/8) contralateral to the target item, and this neural activity shared the typical spatiotemporal characteristics of the N2pc. The N2pc has been associated with visual search and spatial selection of targets among distractors in incoming percepts (e.g., Eimer, 1996; Luck et al., 1997; Hopf et al., 2000; Hickey et al., 2009) as well as with search and detection of targets held in VWM (e.g., Kuo et al., 2009; Dell’Acqua et al., 2010; Shimi et al., 2014a). Obtaining an N2pc here suggests that selective attention during VWM encoding both in childhood and in adulthood involves spatially selecting the target item from the memory array for later recognition. In combination with recent findings where preparatory shifts of attentional orienting did not elicit an N2pc (Shimi et al., 2014a), our current result is consistent with past adult studies suggesting that the N2pc does not simply index the generalized attentional deployment in visual space towards anticipated target locations, but rather it reflects spatial attentional selection of target objects (Kiss et al., 2008; Woodman et al., 2009). The current study extends these observations to the VWM domain both for childhood and adulthood. Following top-down modulations from fronto-parietal areas during preparatory orienting of attention (Murray et al., 2011; Eimer, 2014a,b; Shimi et al., 2014a), the prioritization of a visual percept during encoding in VWM seems to include sensory regions of visual cortex, with posterior parietal occipital cortex coding the attended percept more specifically. This finding is noteworthy for developmental cognitive neuroscientists studying attention and/or VWM, as no prior study has examined the temporal dynamics involved in children’s attentional selection during VWM encoding. Although a few other developmental studies have examined gating mechanisms in VWM (Sander et al., 2011; Astle et al., 2014), these have been focused on a subsequent ERP component to N2pc, i.e., the contralateral delay activity (CDA) which has mainly been investigated in the context of modulation by the number of items currently maintained in VWM, and not in terms of the deployment of selective attention to a specific stimulus for encoding.

Even though the N2pc was elicited in both age groups, there were latency and duration differences of the ERP component between the two age groups; that is, children as a group elicited the N2pc later and for longer than adults. This finding suggests that, although both children and adults can select the target item among multiple competing items during encoding in VWM, at least when appropriate attentional cues that guide selective attention are provided, the two age groups nonetheless differ in their ability to do so. It seems that, at the group level, children are slower and need more time to selectively and efficiently encode the relevant item from irrelevant information in VWM compared to adults. This result is in line with findings from the sensory domain that have shown that the speed and the efficiency of selection for relevant stimulus among competing items improves with age (Ridderinkhof and van der Stelt, 2000). Therefore our result extends previous findings relating selective attention for perception to the VWM domain. This neural change in attentional efficiency during encoding in VWM from childhood to adulthood may be the outcome of richer myelination of axons taking place across development, which may have an effect on axonal transmission and subsequently on the speed and efficiency of cognitive processing (Giedd et al., 1999; Klingberg et al., 1999; Casey et al., 2005; Craik and Bialystok, 2006).

Finally, despite the reliable presence of the N2pc in children at the group level (which provided a clear neural index of their ability to focus attention and to select the target item for encoding in VWM and for later recognition), our second key finding is the high degree of variability across children in the ability to attend to and encode targets in VWM. Children who demonstrated an “adult-like” neural modulation during the encoding phase of the target item, benefitted the most from attention cues that pointed to the item to-be-probed, and thus to the item that they should encode in VWM. In other words, high-capacity children who elicited the N2pc sharing the same spatio-temporal characteristics of the ERP component observed in adults (i.e., the N2pc was elicited earlier and for shorter period of time) showed a large attention benefit effect in their recognition memory performance for cued trials, compared to neutral trials. In contrast, low-capacity children did not show a robust differentiation in the adult N2pc time window, and showed a small attention benefit in behavioral terms. It is well accepted now that younger and older adults’ ability to regulate access to VWM in a goal-directed manner is vital for protecting VWM capacity from irrelevant information (Vogel and Machizawa, 2004; Gazzaley et al., 2005; Vogel et al., 2005; McNab and Klingberg, 2008; Zanto and Gazzaley, 2009; Murray et al., 2011). Extending recent developmental neuroscience findings that have shown that individual differences in preparatory neural activity prior to encoding information in VWM distinguish children with high vs. low VWM capacity (Shimi et al., 2014a), our current findings demonstrate that individual differences in neural activity underlying selective attention during VWM encoding also discriminate children of high vs. low VWM capacity. Children’s ability to deploy selective attention and to encode only the relevant item in VWM, which ultimately results in higher VWM capacity, is mediated by faster and more efficient neural processing that approximates the adults’ neural profile. Future directions may include the investigation of possible other behavioral correlates of adult-like selection markers: for example, it is possible that children with higher VWM capacity and N2pc also score highly on measures of intelligence, although we did not measure these here. Nonetheless, to our knowledge, this is the first study to show correlations between the mechanisms of selective attentional deployment to a specific target item during VWM encoding (N2pc) and VWM capacity in childhood.

In conclusion, current findings demonstrate that efficient deployment of selective attention goes hand in hand with efficient VWM encoding both in childhood and in adulthood. Although behavioral data do not seem sensitive enough to capture age group differences in processing speed of VWM encoding, the underlying neural pattern demonstrates that from childhood children with more refined skills in selective attention, exhibit higher VWM capacity. These findings provide new insights to the relatively recent developmental cognitive neuroscience literature examining attentional contributions to increases in VWM capacity. Future studies examining the developmental trajectories of selective attention in service of VWM capacity can shed light on the maturation of the N2pc and behavioral related parameters.

## Conflict of Interest Statement

The authors declare that the research was conducted in the absence of any commercial or financial relationships that could be construed as a potential conflict of interest.

## References

[B1] AEEGS. (1991). American electroencephalographic society guidelines for standard electrode position nomenclature. J. Clin. Neurophysiol. 8, 200–202. 10.1097/00004691-199104000-00007 2050819

[B2] Astle D. E. Harvey H. Stokes M. Mohseni H. Nobre A. C. Scerif G. (2014). Distinct neural mechanisms of individual and developmental differences in VSTM capacity. Dev. Psychobiol. 56, 601–610. 10.1002/dev.21126 23775219

[B3] Astle D. E. Nobre A. C. Scerif G. (2012). Attentional control constrains visual short-term memory: insights from developmental and individual differences. Q. J. Exp. Psychol. Hove. 65, 277–294. 10.1080/17470218.2010.492622 20680889 PMC4152725

[B4] Astle D. E. Scerif G. Kuo B. C. Nobre A. C. (2009). Spatial selection of features within perceived and remembered objects. Front. Human Neurosci. 3:6. 10.3389/neuro.09.006.2009 19434243 PMC2679200

[B5] Barriga-Paulino C. I. Flores A. B. Gómez C. M. (2011). Developmental changes in the eeg rhythms of children and young adults. J. Psychophysiol. 25, 143–158. 10.1027/0269-8803/a000052

[B6] Barriga-Paulino C. I. Rodríguez-Martínez E. I. Rojas-Benjumea M. Á. Gómez C. M. (2014). Slow wave maturation on a visual working memory task. Brain Cogn. 88, 43–54. 10.1016/j.bandc.2014.04.003 24859090

[B7] Casey B. J. Tottenham N. Liston C. Durston S. (2005). Imaging the developing brain: what have we learned about cognitive development? Trends Cogn. Sci. 9, 104–110. 10.1016/j.tics.2005.01.011 15737818

[B8] Chun M. M. Johnson M. K. (2011). Memory: enduring traces of perceptual and reflective attention. Neuron 72, 520–535. 10.1016/j.neuron.2011.10.026 22099456 PMC3248396

[B9] Cohen J. R. Sreenivasan K. K. D’Esposito M. (2014). Correspondence between stimulus encoding-and maintenance-related neural processes underlies successful working memory. Cereb. Cortex 24, 593–599. 10.1093/cercor/bhs339 23146963 PMC3920762

[B10] Corbetta M. Kincade J. M. Shulman G. L. (2002). Neural systems for visual orienting and their relationships to spatial working memory. J. Cogn. Neurosci. 14, 508–523. 10.1162/089892902317362029 11970810

[B11] Corbetta M. Kincade J. M. Ollinger J. M. McAvoy M. P. Shulman G. L. (2000). Voluntary orienting is dissociated from target detection in human posterior parietal cortex. Nat. Neurosci. 3, 292–297. 10.1038/73009 10700263

[B12] Couperus J. W. Quirk C. (2015). Visual search and the N2pc in children. Atten. Percept. Psychophys. 77, 768–776. 10.3758/s13414-015-0833-5 25678274 PMC4381109

[B13] Cowan N. (2001). The magical number 4 in short-term memory: a reconsideration of mental storage capacity. Behav. Brain Sci. 24, 87–185. 10.1017/s0140525x01003922 11515286

[B14] Cowan N. Elliott E. M. Scott Saults J. Morey C. C. Mattox S. Hismjatullina A.. (2005). On the capacity of attention: its estimation and its role in working memory and cognitive aptitudes. Cogn. Psychol. 51, 42–100. 10.1016/j.cogpsych.2004.12.001 16039935 PMC2673732

[B15] Cowan N. Morey C. C. AuBuchon A. M. Zwilling C. E. Gilchrist A. L. (2010). Seven-year-olds allocate attention like adults unless working memory is overloaded. Dev. Sci. 13, 120–133. 10.1111/j.1467-7687.2009.00864.x 20121868 PMC2819460

[B16] Craik F. I. M. Bialystok E. (2006). Cognition through the lifespan: mechanisms of change. Trends Cogn. Sci. 10, 131–138. 10.1016/j.tics.2006.01.007 16460992

[B17] Crone E. A. Ridderinkhof K. R. (2011). The developing brain: from theory to neuroimaging and back. Dev. Cogn. Neurosci. 1, 101–109. 10.1016/j.dcn.2010.12.001 22436435 PMC6987573

[B18] Dell’Acqua R. Sessa P. Toffanin P. Luria R. Jolicoeur P. (2010). Orienting attention to objects in visual short-term memory. Neuropsychologia 48, 419–428. 10.1016/j.neuropsychologia.2009.09.033 19804791

[B19] Desimone R. (1998). Visual attention mediated by biased competition in extrastriate visual cortex. Philos. Trans. R. Soc. Lond. B. Biol. Sci. 353, 1245–1255. 10.1098/rstb.1998.0280 9770219 PMC1692333

[B20] Desimone R. Duncan J. (1995). Neural mechanisms of selective visual attention. Annu. Rev. Neurosci. 18, 193–222. 10.1146/annurev.neuro.18.1.193 7605061

[B21] Eimer M. (1996). The N2pc component as an indicator of attentional selectivity. Electroencephalogr. Clin. Neurophysiol. 99, 225–234. 10.1016/0013-4694(96)95711-9 8862112

[B22] Eimer M. (2014a). The neural basis of attentional control in visual search. Trends Cogn. Sci. 18, 526–535. 10.1016/j.tics.2014.05.005 24930047

[B23] Eimer M. (2014b). “The time course of spatial attention: insights from event-related brain potentials,” in The Oxford Handbook of Attention, eds Nobre A. Kastner S. (Oxford: Oxford University Press), 289–317.

[B24] Fusser F. Linden D. E. J. Rahm B. Hampel H. Haenschel C. Mayer J. S. (2012). Common capacity-limited neural mechanisms of selective attention and spatial working memory encoding. Eur. J. Neurosci. 34, 827–838. 10.1111/j.1460-9568.2011.07794.x 21781193 PMC3465779

[B25] Gathercole S. E. (1999). Cognitive approaches to the development of short-term memory. Trends Cogn. Sci. Regul. Ed. 3, 410–419. 10.1016/s1364-6613(99)01388-1 10529796

[B26] Gazzaley A. Nobre A. C. (2012). Top-down modulation: bridging selective attention and working memory. Trends Cogn. Sci. 16, 129–135. 10.1016/j.tics.2011.11.014 22209601 PMC3510782

[B27] Gazzaley A. Cooney J. W. Rissman J. D’Esposito M. (2005). Top-down suppression deficit underlies working memory impairment in normal aging. Nat. Neurosci. 8, 1298–1300. 10.1038/nn1543 16158065

[B28] Giedd J. N. Blumenthal J. Jeffries N. O. Castellanos F. X. Liu H. Zijdenbos A.. (1999). Brain development during childhood and adolescence: a longitudinal MRI study. Nat. Neurosci. 2, 861–863. 10.1038/13158 10491603

[B29] Griffin I. C. Nobre A. C. (2003). Orienting attention to locations in internal representations. J. Cogn. Neurosci. 15, 1176–1194. 10.1162/089892903322598139 14709235

[B30] Hickey C. Di Lollo V. McDonald J. J. (2009). Electrophysiological indices of target and distractor processing in visual search. J. Cogn. Neurosci. 21, 760–775. 10.1162/jocn.2009.21039 18564048

[B31] Hillyard S. A. Anllo-Vento L. (1998). Event-related brain potentials in the study of visual selective attention. Proc. Natl. Acad. Sci. U S A 95, 781–787. 10.1073/pnas.95.3.781 9448241 PMC33798

[B32] Hopf J. M. Luck S. J. Girelli M. Hagner T. Mangun G. R. Scheich H.. (2000). Neural sources of focused attention in visual search. Cereb. Cortex 10, 1233–1241. 10.1093/cercor/10.12.1233 11073872

[B33] Hopf J.-M. Boelmans K. Schoenfeld M. A. Luck S. J. Heinze H. J. (2004). Attention to features precedes attention to locations in visual search: evidence from electromagnetic brain responses in humans. J. Neurosci. 24, 1822–1832. 10.1523/jneurosci.3564-03.2004 14985422 PMC6730400

[B34] Huizinga M. Dolan C. V. van der Molen M. W. (2006). Age-related change in executive function: Developmental trends and a latent variable analysis. Neuropsychologia 44, 2017–2036. 10.1016/j.neuropsychologia.2006.01.010 16527316

[B35] Ikkai A. Curtis C. E. (2011). Common neural mechanisms supporting spatial working memory, attention and motor intention. Neuropsychologia 49, 1428–1434. 10.1016/j.neuropsychologia.2010.12.020 21182852 PMC3081523

[B36] Johnson M. H. (2011). “Vision, orienting and attention,” in Developmental Cognitive Neuroscience, edsJohnson M. H. de Haan M. (Hoboken: Wiley-Blackwell), 81–104.

[B37] Jolles D. D. Kleibeuker S. W. Rombouts S. A. R. B. Crone E. A. (2011). Developmental differences in prefrontal activation during working memory maintenance and manipulation for different memory loads. Dev. Sci. 14, 713–724. 10.1111/j.1467-7687.2010.01016.x 21676092

[B38] Jost K. Bryck R. L. Vogel E. K. Mayr U. (2011). Are old adults just like low working memory young adults? Filtering efficiency and age differences in visual working memory. Cereb. Cortex 21, 1147–1154. 10.1093/cercor/bhq185 20884722

[B39] Kastner S. Ungerleider L. G. (2001). The neural basis of biased competition in human visual cortex. Neuropsychologia 39, 1263–1276. 10.1016/s0028-3932(01)00116-6 11566310

[B40] Kiss M. Van Velzen J. Eimer M. (2008). The N2pc component and its links to attention shifts and spatially selective visual processing. Psychophysiology 45, 240–249. 10.1111/j.1469-8986.2007.00611.x 17971061 PMC2248220

[B41] Klingberg T. Forssberg H. Westerberg H. (2002). Training of working memory in children with ADHD. J. Clin. Exp. Neuropsychol. 24, 781–791. 10.1076/jcen.24.6.781.8395 12424652

[B42] Klingberg T. Vaidya C. J. Gabrieli J. D. Moseley M. E. Hedehus M. (1999). Myelination and organization of the frontal white matter in children: a diffusion tensor MRI study. Neuroreport 10, 2817–2821. 10.1097/00001756-199909090-00022 10511446

[B43] Kuo B. C. Rao A. Lepsien J. Nobre A. C. (2009). Searching for targets within the spatial layout of visual short-term memory. J. Neurosci. 29, 8032–8038. 10.1523/jneurosci.0952-09.2009 19553443 PMC6666054

[B44] Kwon H. Reiss A. L. Menon V. (2002). Neural basis of protracted developmental changes in visuo-spatial working memory. Proc. Natl. Acad. Sci. U S A 99, 13336–13341. 10.1073/pnas.162486399 12244209 PMC130634

[B45] Linke A. C. Vicente-Grabovetsky A. Mitchell D. J. Cusack R. (2011). Encoding strategy accounts for individual differences in change detection measures of VSTM. Neuropsychologia 49, 1476–1486. 10.1016/j.neuropsychologia.2010.11.034 21130789

[B46] Luck S. J. Hillyard S. A. (1994a). Electrophysiological correlates of feature analysis during visual search. Psychophysiology 31, 291–308. 10.1111/j.1469-8986.1994.tb02218.x 8008793

[B47] Luck S. J. Hillyard S. A. (1994b). Spatial filtering during visual search: evidence from human electrophysiology. J. Exp. Psychol. Hum. Percept. Perform. 20, 1000–1014. 10.1037/0096-1523.20.5.1000 7964526

[B48] Luck S. J. Girelli M. McDermott M. T. Ford M. A. (1997). Bridging the gap between monkey neurophysiology and human perception: an ambiguity resolution theory of visual selective attention. Cogn. Psychol. 33, 64–87. 10.1006/cogp.1997.0660 9212722

[B49] Luck S. J. Woodman G. F. Vogel E. K. (2000). Event-related potential studies of attention. Trends Cogn. Sci. 4, 432–440. 10.1016/s1364-6613(00)01545-x 11058821

[B50] Luna B. Garver K. E. Urban T. A. Lazar N. A. Sweeney J. A. (2004). Maturation of cognitive processes from late childhood to adulthood. Child Dev. 75, 1357–1372. 10.1111/j.1467-8624.2004.00745.x 15369519

[B51] Macmillan N. A. Creelman C. D. (2005). Detection Theory: A User’s Guide 2nd ed. (Mahwah, NJ: Lawrence Erlbaum Associates).

[B52] Markant J. Amso D. (2013). Selective memories: infants’ encoding is enhanced in selection via suppression. Dev. Sci. 16, 926–940. 10.1111/desc.12084 24118717 PMC3801267

[B53] Mayer J. S. Bittner R. A. Nikolić D. Bledowski C. Goebel R. Linden D. E. J. (2007). Common neural substrates for visual working memory and attention. Neuroimage 36, 441–453. 10.1016/j.neuroimage.2007.03.007 17462914

[B54] Mazza V. Turatto M. Caramazza A. (2009). Attention selection, distractor suppression and N2pc. Cortex 45, 879–890. 10.1016/j.cortex.2008.10.009 19084218

[B55] McNab F. Klingberg T. (2008). Prefrontal cortex and basal ganglia control access to working memory. Nat. Neurosci. 11, 103–107. 10.1038/nn2024 18066057

[B56] Melinder A. Gredeback G. Westerlund A. Nelson C. A. (2010). Brain activation during upright and inverted encoding of own- and other-age faces: ERP evidence for an own-age bias. Dev. Sci. 13, 588–598. 10.1111/j.1467-7687.2009.00910.x 20590723 PMC2898522

[B57] Murray A. M. Nobre A. C. Stokes M. G. (2011). Markers of preparatory attention predict visual short-term memory performance. Neuropsychologia 49, 1458–1465. 10.1016/j.neuropsychologia.2011.02.016 21335015 PMC3318119

[B58] Nobre A. C. Griffin I. C. Rao A. (2008). Spatial attention can bias search in visual short-term memory. Front. Hum. Neurosci. 1:4. 10.3389/neuro.09.004.2007 18958218 PMC2525979

[B59] Olesen P. J. Macoveanu J. Tegnér J. Klingberg T. (2007). Brain activity related to working memory and distraction in children and adults. Cereb. Cortex 17, 1047–1054. 10.1093/cercor/bhl014 16801377

[B60] Pashler H. (1988). Familiarity and visual change detection. Percept. Psychophys. 44, 369–378. 10.3758/bf03210419 3226885

[B61] Plude D. J. Enns J. T. Brodeur D. (1994). The development of selective attention: A life-span overview. Acta. Psychol. Amst. 86, 227–272. 10.1016/0001-6918(94)90004-3 7976468

[B62] Ridderinkhof K. R. van der Stelt O. (2000). Attention and selection in the growing child: views derived from developmental psychophysiology. Biol. Psychol. 54, 55–106. 10.1016/s0301-0511(00)00053-3 11035220

[B63] Ross-Sheehy S. Oakes L. M. Luck S. J. (2011). Exogenous attention influences visual short-term memory in infants. Dev. Sci. 14, 490–501. 10.1111/j.1467-7687.2010.00992.x 21477189 PMC3076103

[B64] Sander M. C. Werkle-Bergner M. Lindenberger U. (2011). Contralateral delay activity reveals life-span age differences in top-down modulation of working memory contents. Cerebral Cortex 21, 2809–2819. 10.1093/cercor/bhr076 21527784

[B65] Scerif G. (2010). Attention trajectories, mechanisms and outcomes: at the interface between developing cognition and environment. Dev. Sci. 13, 805–812. 10.1111/j.1467-7687.2010.01013.x 20977552

[B66] Scerif G. Kotsoni E. Casey B. J. (2006). “The functional neuroimaging of development,” in Functional NeuroImaging of Cognition, eds Cabeza R. Kingstone A. (Cambridge, MA: MIT Press), 351–378.

[B68] Shimi A. Kuo B. C. Astle D. E. Nobre A. C. Scerif G. (2014a). Age group and individual differences in attentional orienting dissociate neural mechanisms of encoding and maintenance in visual STM. J. Cogn. Neurosci. 26, 864–877. 10.1162/jocn_a_00526 24236697

[B69] Shimi A. Nobre A. C. Astle D. Scerif G. (2014b). Orienting attention within visual short-term memory: development and mechanisms. Child Dev. 85, 578–592. 10.1111/cdev.12150 23937596

[B67] Shimi A. Scerif G. (2015). The interplay of spatial attentional biases and mental codes in VSTM: developmentally informed hypotheses. Dev. Psychol. 51, 731–743. 10.1037/a0039057 25844847

[B70] Stevens C. Bavelier D. (2012). The role of selective attention on academic foundations: A cognitive neuroscience perspective. Dev. Cogn. Neurosci. 2, S30–S48. 10.1016/j.dcn.2011.11.001 22682909 PMC3375497

[B71] Todd J. J. Marois R. (2004). Capacity limit of visual short-term memory in human posterior parietal cortex. Nature 428, 751–754. 10.1038/nature02466 15085133

[B72] Vogel E. K. Machizawa M. G. (2004). Neural activity predicts individual differences in visual working memory capacity. Nature 428, 748–751. 10.1038/nature02447 15085132

[B73] Vogel E. K. McCollough A. W. Machizawa M. G. (2005). Neural measures reveal individual differences in controlling access to working memory. Nature 438, 500–503. 10.1038/nature04171 16306992

[B74] Wendelken C. Baym C. L. Gazzaley A. Bunge S. A. (2011). Neural indices of improved attentional modulation over middle childhood. Dev. Cogn. Neurosci. 1, 175–186. 10.1016/j.dcn.2010.11.001 21516182 PMC3080660

[B75] Woodman G. F. Arita J. T. Luck S. J. (2009). A cuing study of the N2pc component: an index of attentional deployment to objects rather than spatial locations. Brain Res. 1297, 101–111. 10.1016/j.brainres.2009.08.011 19682440 PMC2758329

[B76] Woodman G. F. Luck S. J. (1999). Electrophysiological measurement of rapid shifts of attention during visual search. Nature 400, 867–869. 10.1038/23698 10476964

[B77] Zanto T. P. Gazzaley A. (2009). Neural suppression of irrelevant information underlies optimal working memory performance. J. Neurosci. 29, 3059–3066. 10.1523/jneurosci.4621-08.2009 19279242 PMC2704557

